# On a Visual Phenomenon and Its Explanation

**Published:** 1880-05

**Authors:** 


On a Visual Phenomenon and its Explanation.—The phenomenon in question may be seen under the following circumstances: Face the breeze, and without winking allow a small rain-drop to fall on the surface of the cornea, all the while keeping your gaze fixed on a lamp-light some hundred feet away. As the rain-drop alights on the cornea several rings of light appear to surround the luminous source, and *they gradually contract in diameter.*

*Explanation*—In sunshine the moving ring-crest of water produced by dropping a pebble into a still and shallow pool projects a ring of light on the bottom, which gradually increases in size. The moving ring-crest, by its refractive action, produces a hollow cylinder of rays of ever-increasing diameter, and we see a section of it on the bottom of the pool. The rain-drop falling on the cornea spreads out on its surface in several ring-crests, and would similarly produce a series of outward-traveling rings of light were it not for the combined action of the refractive media of the eye. Under the influence of these, two hollow cones of light are formed within the vitreous humor directly upon impact of the rain-drop. The first of these has for its base a small circular area of the hind surface of the lens, and its prolongation: the second cone has the retina for its base. As any individual ring-crest spreads out on the cornea, the first cone increases in size, the common apex advances toward the retina, and consequently the section of the second cone projected on to the retina decreases in size, and appears as a contracting ring of light. (Abstract of a paper by Wm. Ackroyd, F. I. C., read before the members of the British Association, 1879, in Section D. Biology, Department of Anatomy and Physiology.)—*Edinburgh Medical Journal.*

